# Genome-Wide Investigation of WRKY Transcription Factors Involved in Terminal Drought Stress Response in Common Bean

**DOI:** 10.3389/fpls.2017.00380

**Published:** 2017-03-23

**Authors:** Jing Wu, Jibao Chen, Lanfen Wang, Shumin Wang

**Affiliations:** ^1^Key Laboratory of Crop Gene Resources and Germplasm Enhancement, Ministry of Agriculture/The National Key Facility for Crop Gene Resources and Genetic Improvement/Institute of Crop Science, Chinese Academy of Agricultural Sciences,Beijing, China; ^2^College of Agricultural Engineering, Nanyang Normal University,Nanyang, China

**Keywords:** common bean, WRKY transcription factor, drought, qRT-PCR, genome sequence

## Abstract

WRKY transcription factor plays a key role in drought stress. However, the characteristics of the WRKY gene family in the common bean (*Phaseolus vulgaris* L.) are unknown. In this study, we identified 88 complete WRKY proteins from the draft genome sequence of the “G19833” common bean. The predicted genes were non-randomly distributed in all chromosomes. Basic information, amino acid motifs, phylogenetic tree and the expression patterns of PvWRKY genes were analyzed, and the proteins were classified into groups 1, 2, and 3. Group 2 was further divided into five subgroups: 2a, 2b, 2c, 2d, and 2e. Finally, we detected 19 WRKY genes that were responsive to drought stress using qRT-PCR; 11 were down-regulated, and 8 were up-regulated under drought stress. This study comprehensively examines WRKY proteins in the common bean, a model food legume, and it provides a foundation for the functional characterization of the WRKY family and opportunities for understanding the mechanisms of drought stress tolerance in this plant.

## Introduction

The average grain yield of common bean in China is approximately 1,200 kg ha^-1^, which is much lower than its potential yields (FAO, 2014). This low productivity is due to biotic and abiotic stress in the farmer’s field, such as diseases, insects, and especially drought (Wu et al., 2016). The main common bean planting areas in China, i.e., Heilongjiang, Shanxi, and Guizhou Provinces, have suffered from drought in recent years (Piao et al., 2010). Therefore, there is an urgent need to discover new genes to enhance drought tolerance through molecular breeding.

Transcription factors (TFs) are an important class of genes and include NAM, ATAF1/2, and CUC2 (NAC), basic/helix-loop-helix (bHLH), myeloblastosis (MYB), dehydration responsive element binding protein (DREB), APETALA2/ethyleneresponsive element binding factor (AP2/ERF), and WRKY, among others (Lata et al., 2014; Mao et al., 2015; Dossa et al., 2016; He G.H. et al., 2016; Jin et al., 2016; Zhang et al., 2016). TFs are involved in responses to many types of abiotic and biotic stresses, including drought. The TFs involved in the drought response include ZMNAC111 (maize), TaNAC67 (wheat), TaNAC2 (wheat), OsMYB48-1 (rice), OsDREB2A (rice), TaMYBsm1 (wheat), OsAP21 (rice), OsGRAS23 (rice), and others (Cui et al., 2011; Mao et al., 2012, 2014, 2015; Jin et al., 2013; Xiong et al., 2014; Xu et al., 2015; Li M.J. et al., 2016). In the first comprehensive review of the WRKY protein, SPF1 was reported to have been cloned from sweet potato 22 years ago. Since then, many WRKY genes have been found in multiple species, including *Arabidopsis*, rice, wheat, and soybean (Ishiguro and Nakamura, 1994; Ren et al., 2010; Wang F. et al., 2015; Wang et al., 2016; He Y. et al., 2016; Li W. et al., 2016; Yang et al., 2016). PlantTFDB (V4.0) contains 14,549 WRKY genes from 166 species. The species with the most WRKY genes are *Glycine max* (296), *Brassica napus* (285), *Panicum virgatum* (275), *Zoysia matrella* (269), and *Gossypium hirsutum* (238). In contrast, 39 species, including *Coffea canephora* (49), *Genlisea aurea* (38), and *Carica papaya* (49), have fewer than 50 WRKY loci as reported in PlantTFDB.

It is well known that WRKY proteins contain the highly conserved 60 AA WRKY domains (Eulgem et al., 2000; Xie et al., 2005). However, the WRKY amino acid sequences are also replaced by WSKY, WVKY, WKRY, or WKKY in a few WRKY proteins (Xie et al., 2005; Song et al., 2016b). Except for the WRKY domain, WRKY proteins contain zinc finger-like motifs at the C-termini, and the structure is either Cx_4−5_Cx_22−23_HxH or Cx_7_Cx_23_HxC (Eulgem et al., 2000). WRKY proteins can be divided into three groups. Group I contains two WRKY domains and two zinc finger motifs (Cx_4−5_Cx_22−23_HxH), group II has one WRKY domain and one zinc finger motif (Cx_4−5_Cx_22−23_HxH) and is divided into five subgroups, and group III has one WRKY domain containing one zinc finger motif (Cx_7_Cx_23_HxC) (Rushton et al., 2010; Chen et al., 2012).

Many reports have highlighted the involvement of WRKY proteins in seed development, seed dormancy, seed germination, senescence, development, and biotic and abiotic stress responses (Hinderhofer and Zentgraf, 2001; Robatzek and Somssich, 2001; Johnson et al., 2002; Sun et al., 2003; Zhang et al., 2004; Luo et al., 2005; Eulgem, 2006; Zhou et al., 2008; Li et al., 2009; Popescu et al., 2009; Qiu and Yu, 2009; Tao et al., 2009). Here, we focus on the functional analysis of WRKY proteins in response to abiotic stresses, such as drought. Multiple studies have shown that WRKY genes respond to drought. For example, in *Arabidopsis*, AtWRKY25, AtWRKY33, AtWRKY46, AtWRKY57, and AtWRKY63 play key roles in the responses to drought stress (Qiu and Yu, 2009; Wu et al., 2009; Song et al., 2010; Ding et al., 2014). Similarly, in rice, overexpression of OsWRKY11, OsWRKY45, and OsWRK72 results in enhanced drought tolerance (Qiu and Yu, 2009; Wu et al., 2009; Song et al., 2010; Ding et al., 2014). In other crops, HvWRKY38, TaWRKY1, TaWRKY33, TaWRKY44, and TaWRKY93 are also involved in the drought response (Marè et al., 2004; Qin et al., 2015; Wang X. et al., 2015). However, compared the grass family, the action of WRKY proteins in legumes is limited. Plants that overexpress GmWRKY54 show enhanced drought tolerance (Zhou et al., 2008).

WRKY proteins have been studied extensively in a variety of plant species (Ishiguro and Nakamura, 1994; Wu et al., 2005; Li et al., 2009, 2011; Lata et al., 2014; Song et al., 2014, 2016b; He Y. et al., 2016; Wang et al., 2016; Yang et al., 2016). It is important to identify various TF gene families from whole genome sequences, and many have been identified this way, such as the auxin/indole-3-acetic acid gene family from wheat, AP2/ERF and MYB from foxtail millet, NAC from the common bean, and WRKY from peanut (Lata et al., 2014; Muthamilarasan et al., 2014; Qiao et al., 2015; Wu et al., 2016; Song et al., 2016b). However, a comprehensive view of WRKY proteins in the common bean is still lacking. Recently, the whole genome sequences of the common bean (Andean and Mesoamerican gene pools) have been released, and they are an important resource for the genome-wide analysis of WRKY proteins (Schmutz et al., 2014; Vlasova et al., 2016).

In this study, we performed a genome-wide identification of WRKY TFs in the common bean and analyzed their gene structure, genome distribution, conserved motifs and expression patterns under drought stress in detail. We identified a series of potential candidate WRKY genes related to drought tolerance for future analyses of gene function in the common bean.

## Materials and Methods

### Plant Material

In this study, two common bean cultivars, Long 22-0579 (drought-tolerant genotype) and Naihua (drought-sensitive genotype), were used to analyze gene expression patterns under drought stress (Wu et al., 2014). The seeds were obtained from the National Gene Bank (China, Beijing). The seedlings were planted in 23-cm × 18-cm (diameter × depth) plastic pots in a greenhouse under a 14/10-h photoperiod at 25°C (day) and 20°C (night). All pots were irrigated to field capacity until 4 weeks after seeding. Two treatments, terminal drought and optimal irrigation, were applied. For the terminal drought treatment, watering was restricted to 25% of field capacity in the pot media from 5 weeks after seeding. For optimal irrigation, the pots were maintained at field capacity throughout the experiment (Wu et al., 2014).

### Sequence Retrieval and WRKY Gene Identification

Common bean whole genome sequences and transcript data were downloaded from the Phytozome genome database (Schmutz et al., 2014). The hidden Markov model (HMM) profile of the WRKY family (PF03106) was extracted from the Pfam database (Finn et al., 2016), and the WRKY HMM profile was used to search the common bean whole genome protein database for the WRKY domain using HMMER 3.0 (Finn et al., 2015). All non-redundant sequences hits with expected values lower than 1E-5 were selected and conserved domain checked using PlantTFDB^1^ and SMART^2^ web server.

### Phylogenetic Analysis

Phylogenetic trees were generated using MEGA 4.1 software, and *cis*-acting regulatory elements (CAREs) were identified using the PlantCARE website^3^ (Lescot et al., 2002). Pairwise non-synonymous substitutions rates (*K*s) and synonymous substitutions rates (*K*a) were calculated using codeml in PAML 4.3a with the F3 × 4 codon frequency model (Yang, 2007). The divergence time was calculated using the formula *T* = *K*s/2λ, assuming a common bean and soybean substitution rate (λ) of 6.1 × 10^-9^ substitutions/synonymous site/year (Egan and Doyle, 2010).

### Analysis of Protein Features

ExPASy was used to determine the basic WRKY gene information [molecular weight (MW), number of amino acids, open reading frame (ORF), ORF length, and isoelectric point (pI)^4^]. Multiple alignment analysis was performed using ClustalW^5^, and subcellular localization was predicted using the softberry website^6^. The structure of the WRKY genes was investigated using the Gene Structure Display Server websites^7^.

### Quantitative RT-PCR

Leaves were obtained from 10 individual plants as they began to wilt under drought stress, immediately frozen in liquid nitrogen and stored at −80°C. Four samples were designated as LOI, LTD, NOI, and NTD according to cultivar (Long 22-0579 [L] or Naihua [N]) and treatment (optimal irrigation [OI] or terminal drought [TD]). Total RNA was extracted using TRIzol reagent (Tiangen, Beijing) according to the manufacturer’s instructions. The quality and quantity of RNA was evaluated by agarose gel electrophoresis and NanoDrop (Thermo Fisher Scientific, Waltham, MA, USA), respectively. For first-strand cDNA synthesis, 1 μg of total RNA after DNA enzyme digestion was synthesized using the SuperScript^®^ II reverse transcriptase kit following the manufacturer’s protocols (Invitrogen, USA).

The qRT-PCR reactions were performed with an ABI PRISM 7300 Sequence Detection System (Thermo Fisher Scientific, Waltham, MA, USA) as follows: 95°C for 30 s followed by 40 cycles of 95°C for 5 s and 60°C for 31 s using SYBR^®^ Premix Ex Taq^TM^ (TaKaRa, Tokyo, Japan). A melting curve was generated as follows: 95°C for 15 s, 60°C for 60 s, and 95°C for 15 s.

All reactions were performed in triplicate, and the relative expression levels were calculated using the 2^-ΔΔCT^ method with normalization to the internal control gene, Skip16 (Borges et al., 2012). The specific gene primers are listed in Supplementary Table S1 and were designed with Primer 5.0 software. In this study, differentially expressed genes with higher expression levels in TD samples than in OI samples were denoted by “up-regulated,” and those with lower expression levels were denoted by “down-regulated.”

## Results

### Identification and Distribution of the WRKY Proteins in the Common Bean

A total of 102 candidate genes corresponding to the Pfam WRKY family were obtained with the HMM (Supplementary Table S2), and 90 non-redundant WRKY genes were identified in the common bean genome, of which 88 full-length protein sequences were used for further analyses (Supplementary Table S3). A total of 88 WRKY proteins were identified from the common bean (G19833) using a bioinformatics approach (Supplementary Table S3). Gene characteristics, including the length of the full-length sequence, length of the CDS, length of the protein sequence, gene MW, pI, subcellular localization and the corresponding positions, were analyzed (Supplementary Table S3). These genes were named PvWRKY1 to PvWRKY88. The length of all full-length sequences ranged from 685 (PvWRKY72) to 7,722 bp (PvWRKY62), with an average of 2,340 bp. The length of the CDS ranged from 450 (PvWRKY23) to 2,223 bp (PvWRKY34), with an average of 1143 bp. The length of the protein sequences ranged from 149 (PvWRKY23) to 740 AA (PvWRKY34), with an average of 380 AA. The gene MW ranged from 17.36 (PvWRKY1) to 80.20 kDa (PvWRKY34), with an average of 42.07 kDa; and the pI ranged from 4.83 (PvWRKY7) to 9.94 (PvWRKY29) with an average of 7.07. The predicted subcellular localization results indicated that 65 PvWRKY proteins were located in the nuclear region, whereas the remaining proteins were located in the extracellular region.

**FIGURE 1:**
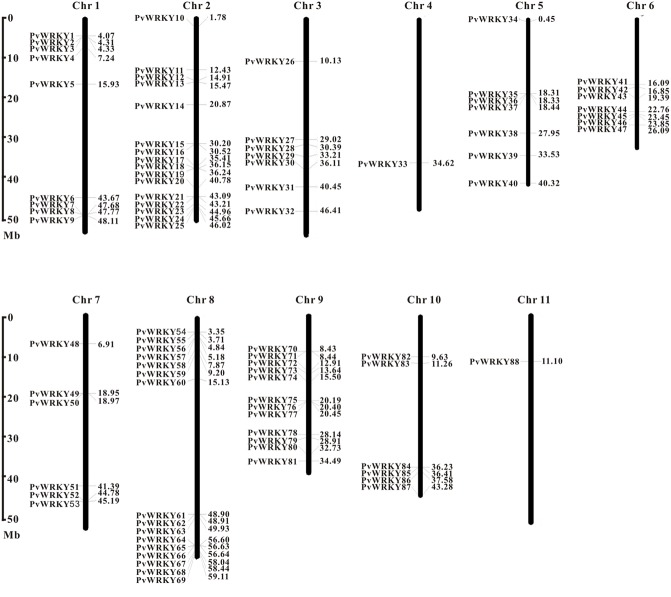
**Chromosomal distribution of common bean WRKY genes.** The position of each PvWRKY gene can be determined using the scale on the left.

**Figure 1** shows that all common bean WRKY genes are distributed across all 11 chromosomes (Ch1–Ch11). The distribution of these genes is uneven. Some chromosomes (e.g., Chr 2 and Chr 8, which represent 18.2% of the WRKY genes) have more genes, whereas others have few (e.g., Chr 9 and Chr 1); some chromosomes have only one gene (Chr 4 and Chr 11); and some of these genes are clustered. To form a cluster, the distance between neighboring WRKY genes had to be less than 200 kb, and separated by no more than eight non-WRKY genes between the WRKY genes (Lozano et al., 2015). We identified 11 small clusters containing 24 WRKY genes, and the average number of WRKY genes in a cluster was 2.2 (Supplementary Table S3). Most of the clusters had two members; only clusters 4 and 8 had three members. Cluster size ranged from 9,066 to 823,695 bp. Cluster 3 was the longest cluster, and cluster 10 was the shortest cluster.

### Multiple Sequence Alignment and Structure Analysis

WRKY proteins contain two very conservative and important motifs, the first of which is the WRKYGQK sequence that always recognizes and binds to the W-box element. In addition to the WRKYGQK sequences, four variants, WRKYGKK, WRKYGEK, WKKYEDK, and WKKYCEDK, were observed in the common bean WRKY proteins (**Supplementary Figure S1** and Table S3). The WRKYGQK sequences represented the major variant in PvWRKY proteins at approximately 90.1%. The second motif is a zinc finger structure containing two types of zinc finger motifs: Cx_4−5_Cx_22−23_HxH and Cx_7_Cx_23_HxC, both of which were observed in the common bean WRKY proteins. Furthermore, 74 PvWRKY proteins contained Cx_4−5_Cx_22−23_HxH-type zinc finger motifs, and 14 PvWRKY proteins contained Cx_7_Cx_23_HxC-type zinc finger motifs (**Supplementary Figure S1** and Table S3).

**FIGURE 2:**
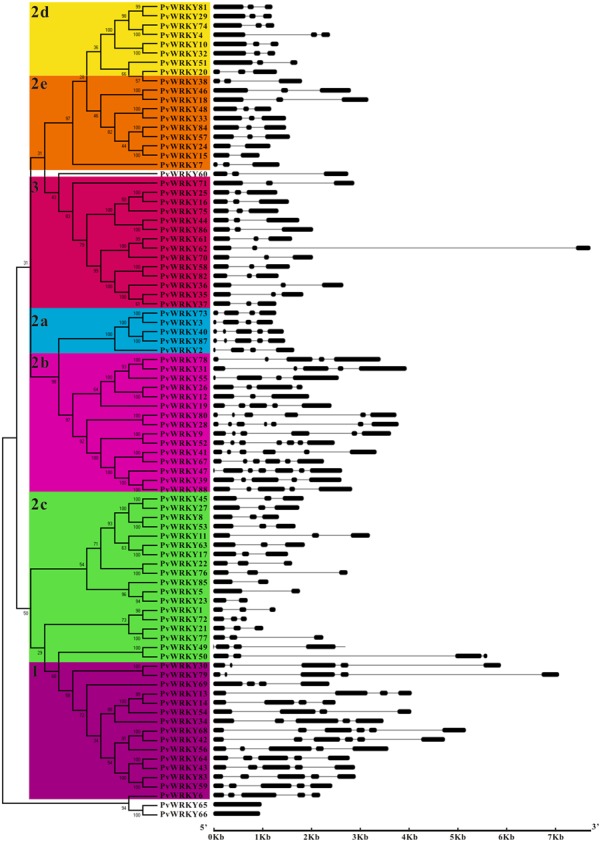
**Phylogenetic tree and gene structure of common bean WRKY genes. (A)** The phylogenetic tree was constructed with MEGA 4.1 software by the neighbor-joining (NJ) method with 1,000 bootstrap replicates. **(B)** Exon/intron structure of PvWRKY genes: the introns and exons are represented by gray lines and black boxes, respectively.

**FIGURE 3:**
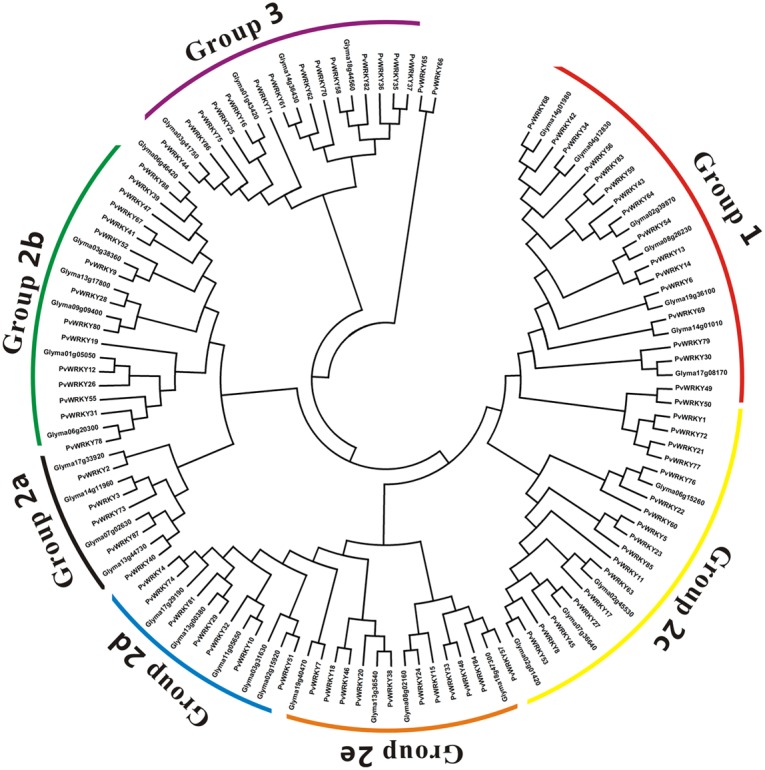
**Phylogenetic tree of PvWRKY proteins and homologous soybean WRKY proteins.** The phylogenetic tree was constructed using same method as used for **Figure 2A**.

The PvWRKY proteins could all be divided into three groups, 1, 2, and 3, containing 15, 57, and 14 proteins, respectively. Notably, PvWRKY65 and PvWRKY66 were not assigned to any group and existed alone because of their unique WRKY sequences (WKKY/CEDK). To better separate the groups, a phylogenetic tree was generated based on the protein sequences of all the PvWRKY genes and 35 homologous soybean WRKY proteins, and the WRKY genes were divided into three groups: 1 (15 sequences), 2 (57 sequences), and 3 (14 sequences) (**Figures 2A**, **3**). Fifty-five sequences from group 2 were further classified into five subgroups: 2a (5 sequences), 2b (15 sequences), 2c (19 sequences), 2d (8 sequences), and 2e (10 sequences). Similar results were also observed in soybean (Song et al., 2016a). Furthermore, each group had a different WRKYGQK sequence and zinc finger motifs; group 1 had two WRKYGQK sequences and two zinc finger motifs. The WRKYGQK sequence of all PvWRKY proteins except PvWRKY 83 from this group was WRKYGQK, and all zinc finger motifs at the N-terminus were CxX_4_Cx_22_Hx_1_H. In contrast, all zinc finger motifs at the C-terminus were Cx_4_Cx_23_Hx_1_H. In group 2, subgroups 2a, 2b, 2d, and 2e had the same motif, WRKYGQK and Cx_5_Cx_23_Hx_1_H. Notably, the WRKYGQK sequence in the subgroup 2c WRKY proteins showed several variations: WRKYGQK was detected in most of the subgroup 2c WRKY proteins; WRKYGKK was detected in PvWRKY1, PvWRKY21, PvWRKY72, and PvWRKY77; and WRKYGEK was detected in PvWRKY23. However, the PvWRKY proteins from group 3 had a consistent motif: WRKYGQK and Cx_7_Cx_23_Hx_1_C.

A detailed illustration of the gene structures is shown in **Figure 2B** and Supplementary Table S3. The number of introns ranged from one to six in all members of the common bean WRKY gene family except PvWRKY65 and PvWRKY66, and the average number of exons among the full-length WRKY genes in the common bean genome was 3.73. Most PvWRKY genes contained the typical splicing of three exons and two introns (45 of the 88 PvWRKY genes); however, PvWRKY28, PvWRKY47, and PvWRKY52 had the greatest numbers, seven exons and six introns, whereas PvWRKY65 and PvWRKY66 each harbored one exon. Overall, the phylogenetic analysis of the PvWRKY gene family showed that genes within the same group generally had a similar structure (**Figure 2B**); for example, subgroups 2d and 2e contained three exons and two introns.

### *Cis*-Acting Regulatory Element Analysis of the WRKY Promoter

We analyzed 1500-bp sequences upstream of the translational start site (Supplementary Table S4) and divided these elements into seven groups: essential element, enhancer, light responsive, tissue-specific expression, abiotic stress, hormone, and other types of elements. In putative essential CAREs, TATA, and CAAT boxes were detected upstream of the transcription start site. The light-responsive type had the highest number of members, harboring 35 elements including Gap-box, AE-box, ATCT-motif, and Box-4. Some CAREs involved in the tissue-specific expression of root, shoot, seed, and meristem were found in the promoter regions of WRKY genes, such as AACA_motif, which is related to endosperm-specific negative expression, and CCGTCC-box, which is involved in meristem-specific activation. The most noteworthy result is that the CAREs associated with abiotic or biotic stress and the related hormones were found in the promoter region; ABRE is involved in abscisic acid responsiveness; P-box, TATC-box, and GARE-motif are gibberellin-responsive elements; TCA-element and SARE are involved in salicylic acid responsiveness; CGTCA-motif and TGACG-motif are involved in MeJA responsiveness; TGA-element and AuxRR-core are involved in auxin responsiveness; MBS is involved in drought inducibility; and DRE and LTR are involved in the responses to dehydration, low temperatures, and salt stress. Furthermore, we observed that more than one abiotic or biotic stress and hormone element occur in one promoter region; for example, PvWRKY67 contained five abiotic or biotic stress elements (LTR, HSE, MBS, WUN-motif, and TC-rich repeats) and four hormone elements (ELI-box3, P-box, CGTCA-motif, TGA-element, and TGACG element), and PvWRKY32 included four abiotic or biotic stress elements (HSE, MBS, WUN-motif, and DRE) and six hormone elements (TCA-element, ABRE, P-box, GARE-motif, CGTCA-motif, and TGACG element).

### Evolutionary Patterns and Divergence of the WRKY Gene Family between the Common Bean and Soybean

The common bean and soybean belong to the legume family and are closely related. In this paper, we determined the *K*a/*K*s substitution ratio in the coding sequences of orthologs between the WRKY families of the common bean and soybean (Supplementary Table S5). All pairwise *K*a/*K*s ratios were below 1 and ranged from 0.0937 to 0.9787, suggesting that the PvWRKY genes are under purifying selection. To confirm the divergence time between the common bean and soybean, divergence times were calculated with relative *K*s values. Most of the *K*s values concentrated at 0.2–0.3, suggesting a large-scale event approximately 16.1–24.2 million years ago (MYA). A previous study estimated that common bean and soybean diverged ∼19.2 MYA (Lavin et al., 2005).

### Transcriptome Atlas Analysis of the WRKY Gene Family

**FIGURE 4:**
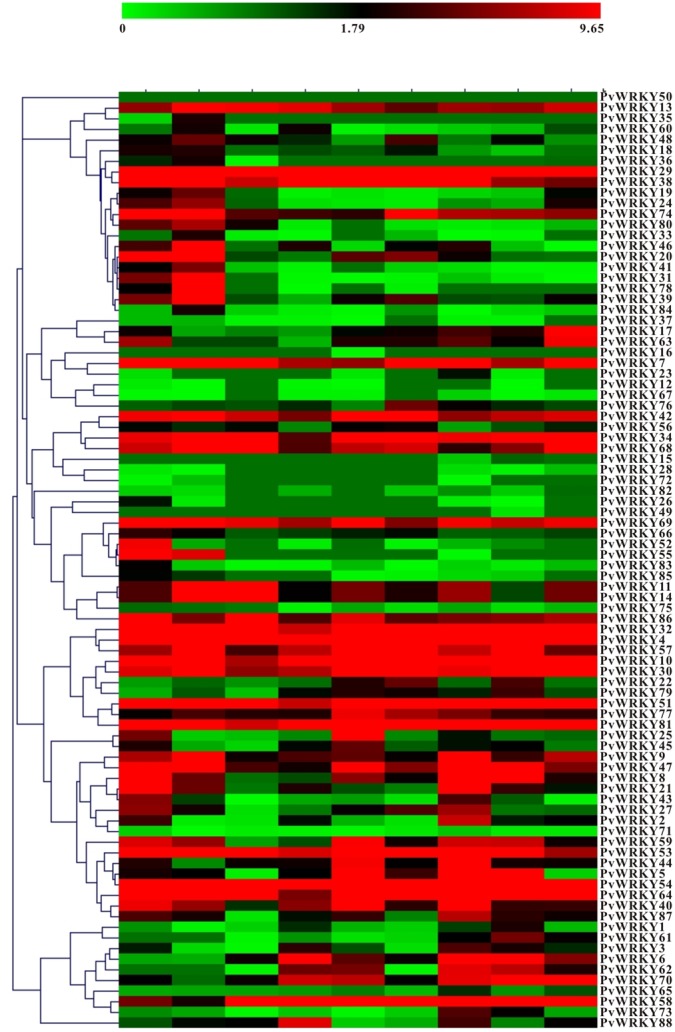
**Heat map of the PvWRKY gene expression profiles in different tissues**.

The availability of transcriptome data facilitates study of the basic biology of the common bean. Expression data were obtained for 87 PvWRKY TFs in nine tissues including young trifoliates, leaves, flower buds, flowers, green mature pods, young pods, roots, stems, and nodules (**Figure 4** and Supplementary Table S6). However, PvWRKY50 lacked expression data. Approximately 93.18% of the PvWRKYs were expressed in nodules, followed by flower and young pods (92.05%), roots (90.91%), and flower buds (86.36%); few WRKY genes were detected in leaves (78.41%). In the common bean, 69.3% (61 of 88) of the PvWRKY proteins were constitutively expressed in every tested tissue. Furthermore, PvWRKY16 and PvWRKY49 were specifically expressed in green mature pods and young pods, respectively. PvWRKY15 was co-expressed in flowers and young pods, whereas PvWRKY35 was co-expressed in nodules and roots. Moreover, PvWRKY4, PvWRKY10, PvWRKY29, PvWRKY32, PvWRKY34, PvWRKY51, PvWRKY54, PvWRKY64, and PvWRKY81 were highly expressed in the vast majority of common bean tissues.

### Expression Profiles of the WRKY Gene Family under Drought Stresses

**FIGURE 5:**
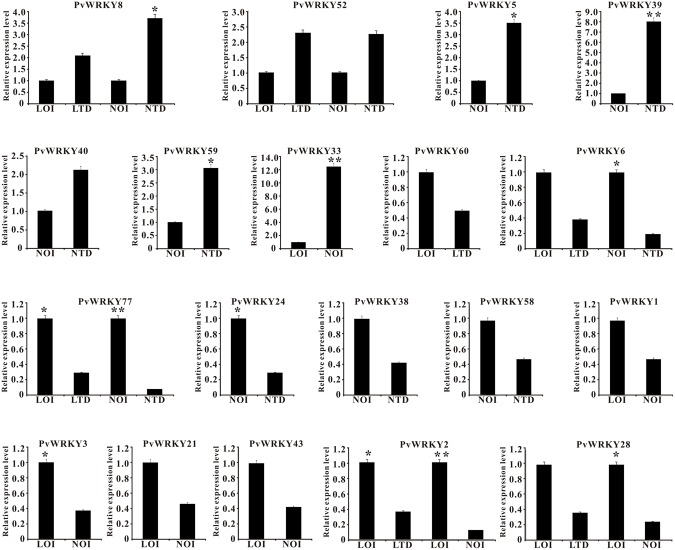
**The expression pattern of PvWRKY genes under drought stress.** The expression values are relative to optimal irrigation conditions (control) for each gene, and the bars represent the averages for the optimally irrigated plants (LOI and NOI) and the drought-stressed plants (LTD and NTD). ^∗^Significantly different at *P* < 0.05; ^∗∗^significantly different at *P* < 0.01.

To determine the expression profiles of the PvWRKY gene family under drought stress, 88 PvWRKY genes were analyzed by qRT-PCR. The expression of the PvWRKY genes was significantly altered (fold change ≥ 2) under drought stress. We obtained 19 PvWRKY genes showing different expression levels under drought stress, including 7 up-regulated and 12 down-regulated genes (**Figure 5** and **Table 1**). Seven WRKY genes showed different expression levels between drought-tolerant and drought-sensitive genotypes. Among these genes, only PvWRKY33 showed a lower expression level in the drought-tolerant genotype than in the drought-sensitive genotype. For the drought-tolerant genotype, PvWRKY8 and PvWRKY52 were up-regulated under drought stress, and five WRKY genes were down-regulated. For the drought-sensitive genotype, six WRKY genes were up-regulated under drought stress, and five were down-regulated. Four genes showed the same expression pattern in the drought-sensitive and drought-tolerant genotypes under drought stress: PvWRKY8 and PvWRKY52 were up-regulated, and PvWRKY6 and PvWRKY77 were down-regulated. PvWRKY2 and PvWRKY28 were down-regulated between LOI/LTD and LOI/NOI.

**Table 1:** The differential expression levels of PvWRKY genes.

Expression pattern		Genes	Fold change (LOI to LTD)	Fold change (NOI to NTD)	Fold change (LOI to NOI)
Up-regulated	LOI/NOI and NOI/NTD	PvWRKY8	2.08	3.67	
		PvWRKY52	2.28	2.25	
	NOI/NTD	PvWRKY5		3.53	
		PvWRKY39		8.14	
		PvWRKY40		2.09	
		PvWRKY59		3.08	
	LOI/NOI	PvWRKY33			12.64
Down-regulated	LOI/LTD	PvWRKY60	−2.02		
	LOI/NOI and NOI/NTD	PvWRKY6	−2.62	−5.39	
		PvWRKY77	−3.41	−12.10	
	NOI/NTD	PvWRKY24		−3.43	
		PvWRKY38		−2.40	
		PvWRKY58		−2.09	
	LOI/NOI	PvWRKY1			−3.33
		PvWRKY3			−2.69
		PvWRKY21			−2.17
		PvWRKY43			−2.33
	LOI/LTD and LOI/NOI	PvWRKY2	−2.71		−8.08
		PvWRKY28	−2.76		−4.07

## Discussion

WRKY proteins have been detected in various organisms, such as spike mosses, single-celled green algae, slime molds and protozoa (Rushton et al., 2010). In monocots and dicots, such as rice, soybean, wheat, and cotton, an especially large number of WRKY proteins have been confirmed to have various functions in recent years (Qiu and Yu, 2009; Luo et al., 2013; Qin et al., 2015; He G.H. et al., 2016; Liu et al., 2016). However, our study is the first to identify and characterize WRKY proteins from whole genome sequences of the common bean.

An increasing number of whole plant genomes have been sequenced, and an increasing number of WRKY genes have been identified in plant species (Yin et al., 2013; He Y. et al., 2016; Song et al., 2016a,b; Wei et al., 2016; Yue et al., 2016). Completion of the common bean genome makes it possible to analyze WRKY TFs at the whole genome level (Schmutz et al., 2014), and in this study, we systematically identified 88 WRKY members in common bean accession G19833. The most obvious variation occurred in CDS length, protein length, PI value, MW, and other basic information, which is consistent with the WRKY family in other plants, such as peanut (Song et al., 2016b). The distributions of the PvWRKY genes across the chromosome appeared to be non-random, as they always formed a gene cluster, which is similar to the results for the WRKY family in other plants and previous genome-wide reports on NAC genes in the common bean (Song et al., 2016a,b; Wu et al., 2016; Yue et al., 2016). Furthermore, in previous studies, WRKY gene numbers varied among species; 74 WRKY proteins have been identified in *A. thaliana* (Ülker and Somssich, 2004), 102 in *Oryza sativa* ssp. *indica* (Ross et al., 2007), 97 in *Oryza sativa* ssp. *japonica* (Rushton et al., 2010), and 75 in *Medicago truncatula* (Rushton et al., 2010). These numbers are similar to the number of WRKY genes in the common bean. In contrast, the number of WRKY genes in soybean (197) (Schmutz et al., 2010) is twice that in the common bean. This difference may be related to species differences in the size of the genome: common bean, 587 Mb (Schmutz et al., 2014); *Arabidopsis*, 119 Mb (The Arabidopsis Genome Initiative, 2000); *Oryza sativa* ssp. *indica*, 466 Mb (Yu et al., 2002); *Oryza sativa* ssp. *japonica*, 420 Mb (Goff et al., 2002); *Medicago truncatula*, 500 Mb (Young et al., 2011); and soybean, 1100 Mb (Schmutz et al., 2010). Therefore, the abundance of WRKY genes has expanded, which may be a result of genome duplications. In the common bean, PvWRKY proteins can be divided into three groups, and group 2 can be divided into five subgroups, 2a, 2b, 2c, 2d, and 2e, based on the amino acid sequences outside the WRKY domain. We also observed that the number of proteins in subgroup 2c was the highest among all subgroups. These results are consistent with the results for other species (Yin et al., 2013; He G.H. et al., 2016; Song et al., 2016a,b; Wei et al., 2016; Yue et al., 2016). However, the variants WRKYGKK, WRKYGEK, WKKYEDK, and WKKYCEDK were mainly observed in subgroup 2c of the common bean, suggesting that the WRKY proteins of subgroup 2c may have a variety of biological functions. The expression profiles revealed different expression patterns for each PvWRKY gene in different tissues, providing a valuable resource for gene functional research. Most PvWRKY genes were expressed in all nine tissues, and nine PvWRKY genes were highly expressed, which suggests that these PvWRKY genes may be essential for plant growth. However, several PvWRKY genes were expressed only in one specific tissue, suggesting that these genes might have tissue-specific functions. The expression profiles generated in this study provide very rich data resources to further investigate the function of PvWRKY genes.

Among the TF families in higher plants, WRKY TFs have been found to play important roles under biotic and abiotic stress, especially drought (Ding et al., 2014; Qin et al., 2015; He Y. et al., 2016; Liu et al., 2016; Yue et al., 2016). Using qRT-PCR in the present study, we identified 19 common bean WRKY TFs that were responsive to drought stress. Among these, 10 PvWRKYs contain MBS elements involved in drought inducibility, such as PvWRKY1, PvWRKY21, PvWRKY28, PvWRKY24, and PvWRKY52. We also found that six PvWRKYs contain ABRE elements involved in drought stress tolerance (Song L. et al., 2016), including PvWRKY2, PvWRKY3, PvWRKY5, PvWRKY38, PvWRKY43, and PvWRKY58. These results support the qRT-PCR results. Furthermore, these genes can be divided two groups: those that were differentially expressed between drought-tolerant and drought-sensitive genotypes and those that were differentially expressed between the treatment and the control. These genes may be good candidates for enhancing drought stress tolerance because we can putatively predict the function of PvWRKY genes based on their identified homologous genes (**Supplementary Figure S2**). For example, GmWRKY27, a PvWRKY40 homolog, improves drought tolerance in transgenic soybean (Wang F. et al., 2015), and GmWRKY54, a PvWRKY53 homolog, confers drought tolerance (Zhou et al., 2008). Interestingly, there have been no reports regarding the functions of several PvWRKY homolog genes, such as PvWRKY5, PvWRKY33, and PvWRKY58; therefore, there may be additional WRKY TFs that are involved in drought resistance in the common bean. However, we also found several PvWRKY genes that were not induced by drought stress but have homologs in other species that function under drought stress. It is possible that the expression of these genes differed only slightly between drought-tolerant and drought-sensitive genotypes. However, different PvWRKYs play different roles in regulating the stress response; therefore, further investigation into their expression patterns under different stresses (salt, heat, and low temperature) is necessary. The results reported here provide some candidates for future studies of the drought resistance mechanism.

In this study, the phylogenetic relationships, exon/intron structures, and expression pattern of WRKY family members under drought stress were evaluated in the common bean, and we identified 19 PvWRKY genes that are responsive to drought stress. As only a few WRKY genes have been detected in the common bean to date, our results will facilitate the functional analysis of PvWRKY genes. These results offer a useful resource for understanding the potential physiological role of individual WRKY genes during drought stress.

## Author Contributions

JW and SW conceived the experiments and analyzed the data, and JC and LW performed the experiments. JW and SW contributed to the writing of the manuscript, which all the authors reviewed.

## Conflict of Interest Statement

The authors declare that the research was conducted in the absence of any commercial or financial relationships that could be construed as a potential conflict of interest.

## Footnotes

1 http://planttfdb.cbi.pku.edu.cn/

2 http://smart.embl-heidelberg.de/

3 http://bioinformatics.psb.ugent.be/webtools/plantcare/html/

4 http://www.expasy.ch/tools/pi_tool.html

5 http://www.genome.jp/tools/clustalw/

6 http://linux1.softberry.com/

7 http://gsds.cbi.pku.edu.cn/

## Supplementary Material

FIGURE S1 **Alignment of multiple common bean WRKY genes**.

FIGURE S2 **Phylogenetic tree of PvWRKY and drought-related WRKY proteins from other species.** The phylogenetic tree was constructed with MEGA4.1 software using the NJ method and 1,000 bootstrap replicates. The green dots represent drought-related WRKY proteins from other species, and the red squares represent drought-related PvWRKY proteins. The accession numbers of all drought-related WRKY proteins follow: TaWRKY93 (JX679079), TaWRKY33 (KT285207), HvWRKY38 (CAD60651), GmWRKY27 (DQ322695), GhWRKY15 (GU207869), GmWRKY54 (DQ322698), AtWRKY57 (AT1G38010), WRKY25 (JF899343), GhWRKY3 (FJ966887), TaWRKY19 (EU665430), OsWRKY30 (ABC02808), TaWRKY2 (EU665425), TaWRKY1 (KT285206), TcWRKY53 (EF053036), AtWRKY63 (AT1G66600), OsWRKY45 (BK065048), AtWRKY46 (AT2G46400), OsWRKY08 LOC_Os05g50610), OsWRKY11 (LOC_Os01g43650), GmWRKY13 (DQ322694), GmWRKY21 (DQ322691).
