# Supplementary material for: Genome-Wide Investigation of WRKY Transcription Factors Involved in Terminal Drought Stress Response in Common Bean
Source: Front Plant Sci. 2017 Mar 23;8:380. doi: 10.3389/fpls.2017.00380 (PMC5362628; doi:10.3389/fpls.2017.00380)
Supplement: FIGURE S1 — Alignment of multiple common bean WRKY genes. [file Image_1.PDF]

# Figure S1 Alignment of multiple common bean WRKY genes

## Group1

```
                WRKYGQ(E)K                           CX4CX22HX1H                           WRKYGQK                          CX4CX23HX1H
PvWRKY6  GYNWRKYGQKQVKGSEYPRSYYKCTQPNCQVKKKVERSHDGQITEIIYKGNHNHVKP.DGFRWRKYGQKVVKGNPYPRSYYRCTNIKCNVRKHVERALDDPRSFVTTYEGKHNHEM
PvWRKY13 GHNWRKYGQKQVKGSEFPRSYYKCTHPHCPVKKKVERSLEGHVTAIIYKGEHNHQRP.DGYRWRKYGQKVVKGNPYPRSYYKCTTPGCKVRKHVERASTDPKAVITTYEGKHNHDV
PvWRKY14 GYNWRKYGQKPVKGSNFSRSYYKCTHPNCPVKKKLELTLQGHVTAIIYKGEHNHQR-.DGYRWRKYGQKVVKGNPYPRSYYKCTTPGCNVRKHVERASTDPKAVVTTYEGKHNHDV
PvWRKY30 GYNWRKYGQKQVKSPTGSRSYYRCTHSDCFAKKIECCDHSGHVIEIVYKSEHSHDPP.DGYRWRKYGQKMVKGNPHPRNYYRCTSAGCPVRKHIESAVDNSDAVIITYKGVHDHDM
PvWRKY34 GYNWRKYGQKQVKGSEYPRSYYKCTHPNCPVKKKVERSFDGQIAEIVYKGEHNHSKP.DGYRWRKYGQKVVRGNPNPRSYYKCTNTGCPVRKHVERASHDPKAVITTYEGKHNHDV
PvWRKY42 GYNWRKYGQKQVKGSEYPRSYYKCTHPNCQVKKKVERSHEGHITEIIYKGTHSHPKP.DGYRWRKYGQKVVRGNPNPRSYYKCTNTGCPVRKHVERASHDPKAVITTYEGKHNHDV
PvWRKY43 GYNWRKYGQKQVKGSENPRSYYKCTYPNCPTKKKVERSLDGQITEIVYKGSHNHPKP.DGYRWRKYGQKVVKGNPNPRSYYKCTFPGCPVRKHVERASHDLRAVITTYEGKHNHDV
PvWRKY54 GFNWRKYGQKQVKGSEYPRSYYKCTHLNCVVKKKVERAPDGHITEIIYKGQHNHEKP.DGYRWRKYGQKVVKGNPHPRSYYKCTSAGCNVRKHVERASTDPKAVITTYEGKHNHDV
PvWRKY56 GYNWRKYGQKHVKGNEFIRSYYKCTYPNCQAKKQLQQSNNGNITDCVSIGQHNHPRP.DGYRWRKYGQKVVKGNPNPRSYYKCTSAGCTVRKHVERASHNLKFVLTTYEGKHNHEV
PvWRKY59 GYNWRKYGQKQVKGSENPRSYYKCTHPNCLVKKKVEMSLDGQITEIVYKGHHNHPKP.DGFRWRKYGQKVVKGNPNARSYYKCTAPGCSVRKHVERAAHDIKAVITTYEGKHNHDV
PvWRKY64 GYNWRKYGQKQVKGSENPRSYYKCTYPNCPTKKKVERSLDGQITEIVYKGTHNHPKP.DGYRWRKYGQKVVKGNPNPRSYYKCTHPGCPVRKHVERASHDLRAVITTYEGKHNHDV
PvWRKY68 GYNWRKYGQKHVKGSEFPRSYYKCTHPNCEVKKLFERSHDGQITEIIYKGTHDHPKP.DGYRWRKYGQKVVRGNPNPRSYYKCTNAGCPVRKHVERASHDPKAVITTYEGKHNHDV
PvWRKY69 GYNWRKYGQKHVKGNEFIRSYYKCTYPNCQAKKQLQQSNNGNITDCVSIGQHNHPRP.DGYRWRKYGQKLVKGNANPRSYYRCSNPGCPVKKHVERASHDSKIVITTYEGQHDHEL
PvWRKY79 GYNWRKYGQKQVKSPTGSRSYYRCTHSNCCAKKIKFCDHSGHVIEIVYKSQHSHDPP.DGYRWRKYGQKLVKGSPHFRNYYRCTSAGCPVRKHIETAVDNTKALIITYKGVHDHDM
PvWRKY83 GYNWRKYGEKQVKGSEHPRSYYKCTYPNCPTKKKVERSLEGHVTEIVYRGSHNHPKP.DGYRWRKYGQKVVKGNPNPRSYYKCVSSGCPVRKHVERAANDMKAVLTTYEGKHNHDV
             WRKY motif                      Zinc-finger structure                  WRKY motif                      Zinc-finger structure
```

## Group 2a

```
                WRKYGQK                                CX5CX23HX1(2)H
PvWRKY2  DGYQWRKYGQKVTKDNASPRAYFRCSMAPICPAKKKVQRCLHDKSILVATYDGEHN-HGA
PvWRKY3  DGYQWRKYGQKVTRDNPSPRAYFKCSFAPSCPVKKKVQRSLEDPTILVTTYEGEHN-HGN
PvWRKY40 DGYQWRKYGQKVTRDNPSPRAYFKCSFAPSCPVKKKVQRSVDDQSVLVATYEGEHN-HPH
PvWRKY73 DGYQWRKYGQKVTRDNPSPRAYFKCSYAPGCPVKKKVQRSVEDPTVLVTTYEGEHN-HGE
PvWRKY87 DGYQWRKYGQKVTRDNPCPRAYFKCSFAPSCPVKKKVQRSVDDQSVLVATYEGEHN-HPQ
```

## Group 2b

```
PvWRKY90 DGCQWRKYGQKMAKGNPCPRAYYRCTMAVGCPVRKQVQRCVEDKTVLTTTYEGNHN-HPL
PvWRKY12 DGCQWRKYGQKMAKGNPCPRAYYRCTVSPLCPVRKQVQRCAEDMSILITTYEGTHN-HPL
PvWRKY19 DGCQWRKYGQKIAKGNPCPRAYYRCTVAPGCPVRKQVQRCIDDMSILITTYEGTHN-HPL
PvWRKY26 DGCQWRKYGQKMAKGNPCPRAYYRCTVSPSCPVKKQVQRCAEDMSILITTYEGTHN-HPL
PvWRKY28 DGCLWRKYGQKISKGNPCPRAYYRCNMGTACPVRKQVQRCATDESVMITTYEGNHN-HSL
PvWRKY31 DGCQWRKYGQKIAKGNPCPRAYYRCTIAPSCPVRKQVQRCVDDMSILITTYEGTHN-HTL
PvWRKY39 DGCQWRKYGQKMAKGNPCPRAYYRCTMAAGCPVRKQVQRCAEDRTILITTYEGNHN-HPL
PvWRKY41 DGCQWRKYGQKMAKGNPCPRAYYRCSMGTGCPVRKQVQRSAEDQSVLITTYEGQHN-HVL
PvWRKY47 DGCQWRKYGQKMAKGNPCPRAYYRCTMAVGCPVRKQVQRCAEDRTILVTTYEGTHN-HPL
PvWRKY52 DGCQWRKYGQKISKGNPCPRAYYRCTMAVGCPVRKQVQRCTEDMSVLITTYEGNHN-HPL
PvWRKY55 DGCQWRKYGQKIAKGNPCPRAYYRCTVAPTCPVRKQVQRCSEDLSILITTYEGTHN-HKL
PvWRKY67 DGCQWRKYGQKMAKGNPCPRAYYRCTMSSACPVRKQVQRCAEDRSVVITTYEGQHN-HAL
PvWRKY78 DGCQWRKYGQKISKGNPCPRAYYRCTVAPSCPVRKQVQRCAQDMTILITTYEGTHN-HPL
PvWRKY80 DGCQWRKYGQKTAKGNPCPRAYYRCSMGTACPVRKQVQRCFKDETVLITTYEGSHN-HPL
PvWRKY88 DGCQWRKYGQKMAKGNPCPRAYYRCTMAAGCPVRKQVQRCAEDRTVLITTYEGNHN-HPL
```

## Group 2d

```
PvWRKY4  DEYSWRKYGQKPIKGSPYPRGYYKCSTVRGCPARKHVERAPDDPAMLIVTYEGEHR-HAV
PvWRKY10 DDYSWRKYGQKPIKGSPHPRGYYKCSSVRGCPARKHVERALDDPAMLVVTYEGEHN-HSL
PvWRKY20 DSWAWRKYGQKPIKGSPYPRGYYRCSSSKGCPARKQVERSCVDPTMLVVTYSSDHN-HPW
PvWRKY29 DEYSWRKYGQKPIKGSPYPRGYYKCSSVRGCPARKHVERAQDDPNMLIVTYEGEHR-HPQ
PvWRKY32 DDYSWRKYGQKPIKGSPHPRGYYKCSSVRGCPARKHVERALDDPAMLVVTYEGEHN-HAL
PvWRKY51 DDYSWRKYGQKPIKGSPHPRGYYKCSSMRGCPARKHVERCLEEPTMLIVTYEGEHN-HPK
PvWRKY74 DEHSWRKYGQKPIKGSPYPRGYYKCSTVRGCPARKHVERASDDPTMLIVTYEGEHR-HSF
PvWRKY81 DEYSWRKYGQKPIKGSPFPRGYYKCSTVKGCPARKHVERAQDDPKMLIVTYEGEHH-HAL
```

## Group 2e

```
PvWRKY7  DFWSWRKYGQKPIKGSPYPRGYYKCSTSKGCSAKKQVERCRTDASTLIITYTSAHN-HPC
PvWRKY15 DPWAWRKYGQKPIKGSPYPRSYYRCSSSKGCLARKFVELSHLDPGVLIVTYTAEHSPHPT
PvWRKY18 DLWAWRKYGQKPIKGSPYPRGYYRCSSSKGCPARKQVERSRTDPNLLVITYTSEHN-HPW
PvWRKY24 DAWAWRKYGQKPIKGSPYPRSYYRCSSSKGCLARKQVERSHLDPAVFLVTYTAEHS-HPH
PvWRKY33 DSWAWRKYGQKPIKGSPYPRNYYRCSSCKGCAARKQVERSTTEPNTFIVTYTGDHK-HAP
PvWRKY38 DSWAWRKYGQKPIKGSPYPRGYYRCSSSKGCPARKQVERSRVDPTKLIVTYANDHN-HSI
PvWRKY46 DLWAWRKYGQKPIKGSPYPRGYYRCSSSKGCSARKQVERSRTDPNMLVITYTSEHN-HPW
PvWRKY48 DLWAWRKYGQKPIKGSPYPRNYYRCSSSKGCMARKQVERSNTETDMFIVTYTGDHS-HPR
PvWRKY57 DIWAWRKYGQKPIKGSPYPRGYYRCSSSKGCLARKQVERNRSDPTMFIVTYTAEHN-HPA
PvWRKY84 DIWAWRKYGQKPIKGSPYPRGYYRCSSSKGCLARKQVERKRSDPTMFIVTYTGEHN-HPA
                 WRKY motif                Zinc-finger structure
```

# Group2c

WRKYG(E)Q(K/D/E)K — $CX_4CX_{22(23)}HX_1H$

```
PvWRKY1   DGYRWRKYGKKMVKNNPNPRNYYRCSVDGCSVKKRVERDKDDPRCVITTYEGSHTHPS
PvWRKY5   DGYRWRKYGQKAVKNNKFPRSYYRCTHQGCNVKKQVQRLTKDEGVVVTTYEGVHTHPI
PvWRKY8   DGYRWRKYGQKAVKNSPFPRSYYRCTSVSCNVKKRVERSFTDPSIVVTTYEGQHTHPS
PvWRKY11  DGYRWRKYGQKAVKNSPFPRSYYRCTNSKCTVKKRVERSSEDPTIVITTYEGQHCHHT
PvWRKY17  DGYRWRKYGQKAVKNSPYPRSYYRCTTQKCTVKKRVERSFQDPTTVITTYEGQHNHPV
PvWRKY21  DGYKWRKYGKKSVKSNPNLRNYYKCSSGGCSVKKRVERDREDSSYVITTYEGVHNHES
PvWRKY22  DGYKWRKYGQKVVKNTQHPRSYYRCTQDNCRVKKRVERLAEDPRMVITTYEGRHVHSP
PvWRKY23  DGYRWRKYGEKSVKNNKFPRSYYRCSYRGCNVKKQIQRHSKDEEIVVTTYEGTHSHPV
PvWRKY27  DGYRWRKYGQKAVKNSPHPRSYYRCTTAACGVKKRVERSSEDPSVVVTTYEGQHTHPC
PvWRKY45  DGYKWRKYGQKAVKNSPYPRSYYRCTTAGCGVKKRVERSSDDPSIVVTTYEGQHTHPC
PvWRKY49  DGYRWRKYGQKTIKGNLFPRAYYKCTSSGCCVRKHVERDSRNRKNVITTYEGKHNHEQ
PvWRKY50  DGYHWRKYGQKPIKGNVFPRAYYKCTTAGCSVKKHVERDSRNQKNLISTYEGRHNHEQ
PvWRKY53  DGYRWRKYGQKAVKNSPFPRSYYRCTSVSCNVKKRVERSFTDPSVVVTTYEGQHTHPS
PvWRKY60  DGYRWRKYGQKVVKGNPNPRSYYKCTSAGCTVRKHVERASHNLKFVLTTYEGKHNHEV
PvWRKY63  DGYRWRKYGQKAVKNSPYPRSYYRCTSQKCSVKKRVERSFQDPSVVITTYEGQHNHHC
PvWRKY72  DGFKWRKYGKKKVKNSPNPRNYYKCSVDGCSVKKTVEREKDDPRYVITTYVGTHTHPS
PvWRKY76  DGYKWRKYGQKVVKNTQHPRSYYRCTQDNCRVKKRVERLAEDPRMVITTYEGRHVHSP
PvWRKY77  DGYKWRKYGKKTVKNNSNPRNYYKCSDEGCIVKKRVERDRDDSSYVLTTYVGIHNHET
PvWRKY85  DGYRWRKYGQKAVKNNMHPRSYYRCTHHTCNVKKQVQRLSKDTSIVVTTYEGIHNHPC
PvWRKY65  DGYNWKKYEDKVGKGNENRVSYYRCTHPNCYAKKKVDR-TIDGKIVEIHYQGNHAHPK
PvWRKY66  EGYNWKKCEDKTGKGNENQVSYYKCTHPNCYVNKKVVR-TIDGKIVEIHYQGNHAHCK
```

WRKY motif — Zinc-finger structure

# Group3

WRKYGQK $CX_{7(6)}CX_{23}HX_{1}C$

```
PvWRKY16 DGYSWRKYGQKKILTAKHPRSYYRCTFSKTTGCWAKKQVQRSEEDPNVFDISYRGSHICYK
PvWRKY25 DGYNWRKYGQKDILGAKYPRSYYRCTFRNTQGCWATKQVQRSDEDPTIFDITYRGKHTCSQ
PvWRKY35 DGYIWRKYGQKMTMNTKYLRSYYRCTHKYDQGCPATKQVQRIQEDPPLYRTTYYGHHNCKI
PvWRKY36 DGYVWRKYGQKMTMNAKYLRSYYRCTHKNDQGCQAMKQVQRIEDDPPLYRTNYYGHHTCKS
PvWRKY37 DGYVWRKYGQKITTNAKYFRSYYRCTHKHDKGCPAIKQVQRIQEDPPLYRTTYYGNHNCKS
PvWRKY44 DGYSWRKYGQKDILGAKFPRGYYRCTHRNVQGCLATKQVQRSDEDPTTIEVTYRGRHTCTQ
PvWRKY58 DGHQWRKYGQKEILNAKFPRNYYRCTHRDQG-CSAKKQVQRVEEDPILYKTIYHGDHTCKS
PvWRKY61 DNYAWRKYGQKEIQNSEFPRSYFRCSYKNDQGCKATKQVQLDHDNPHMYRITYIGIHTCNV
PvWRKY62 DNHAWRKYGQKEIVNSQFPRSYFRCSHKYDQGCQATKQVQRNQECPTMYQTTYTGIHTC--
PvWRKY70 DGFTWRKYGQKEILGSKYPRSYYRCTHQKLYECQAKKMVQRLDHNANIFEVTYRGKHTCHM
PvWRKY71 DNHAWRKYGQKDILNSQFPRSYFRCTRKFEQGCKAMKQVQRLEENPDMYNITYIGLHTCKD
PvWRKY75 DGYSWRKYGQKDILGAMYPRGYYRCTHRTVQGCMATKQVQRSDEDSTIFEINYRGKHTCTV
PvWRKY82 DGHHWRKYGQKEILKTKYSRNYYRCTHKYDGNCQATKQVQRIQEDPPLYKTTYFGHHTCND
PvWRKY86 DGYSWRKYGQKDILGAKFPRAYYRCTYRNIQGCLATKQVQKSDEDPMVNEITYIGRHTCTQ
```

WRKY motif Zinc-finger structure
