# Supplementary figures and images for: Genome-Wide Investigation of WRKY Transcription Factors Involved in Terminal Drought Stress Response in Common Bean
Source: Front Plant Sci. 2017 Mar 23;8:380. doi: 10.3389/fpls.2017.00380 (PMC5362628; doi:10.3389/fpls.2017.00380)

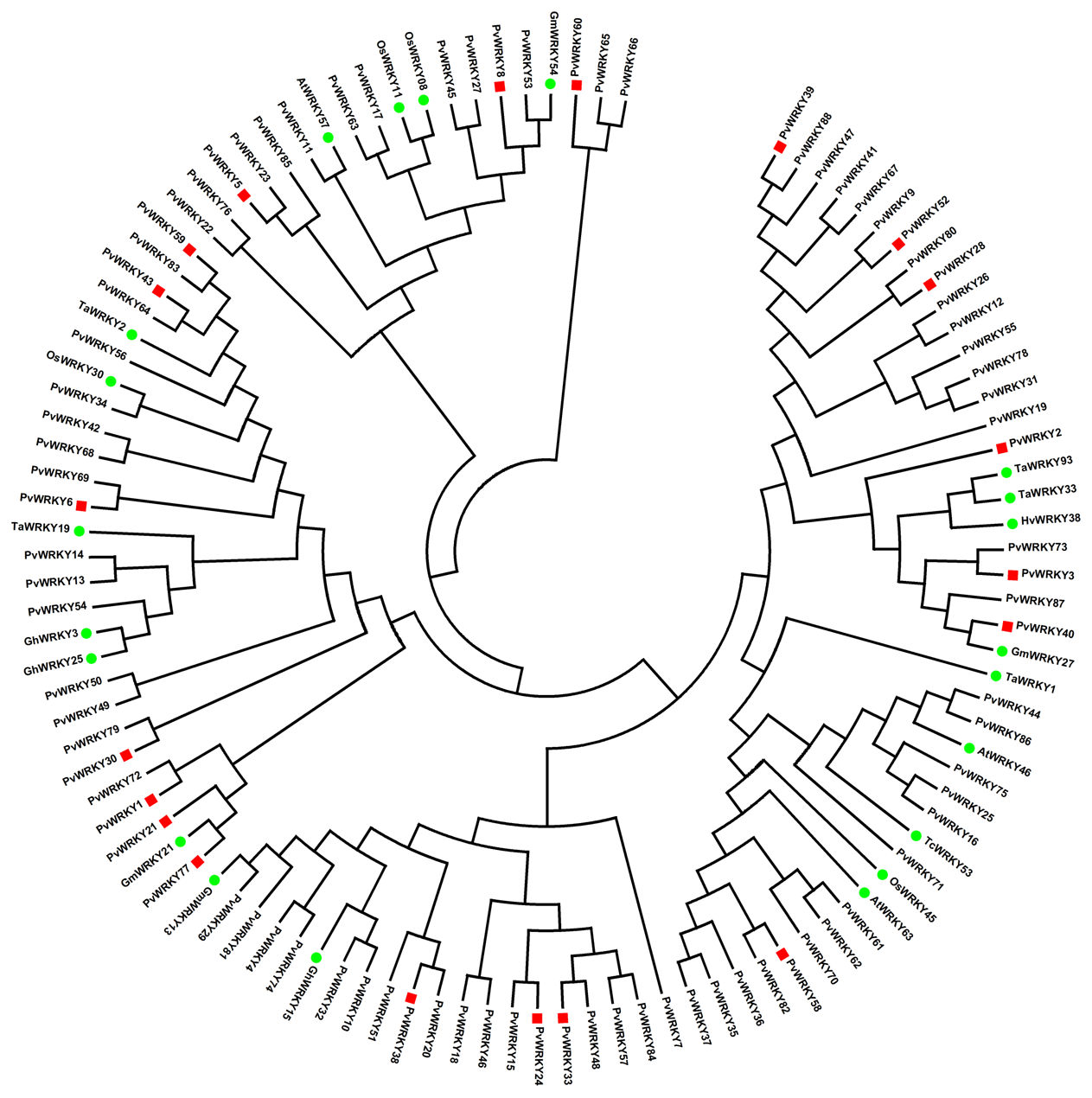

Supplement: FIGURE S2 — Phylogenetic tree of PvWRKY and drought-related WRKY proteins from other species. The phylogenetic tree was constructed with MEGA4.1 software using the NJ method and 1,000 bootstrap replicates. The green dots represent drought-related WRKY proteins from other species, and the red squares represent drought-related PvWRKY proteins. The accession numbers of all drought-related WRKY proteins follow: TaWRKY93 (JX679079), TaWRKY33 (KT285207), HvWRKY38 (CAD60651), GmWRKY27 (DQ322695), GhWRKY15 (GU207869), GmWRKY54 (DQ322698), AtWRKY57 (AT1G38010), WRKY25 (JF899343), GhWRKY3 (FJ966887), TaWRKY19 (EU665430), OsWRKY30 (ABC02808), TaWRKY2 (EU665425), TaWRKY1 (KT285206), TcWRKY53 (EF053036), AtWRKY63 (AT1G66600), OsWRKY45 (BK065048), AtWRKY46 (AT2G46400), OsWRKY08 LOC_Os05g50610), OsWRKY11 (LOC_Os01g43650), GmWRKY13 (DQ322694), GmWRKY21 (DQ322691). [file Image_2.TIF]
